# Metabolomics Reveals Amino Acids Contribute to Variation in Response to Simvastatin Treatment

**DOI:** 10.1371/journal.pone.0038386

**Published:** 2012-07-09

**Authors:** Miles Trupp, Hongjie Zhu, William R. Wikoff, Rebecca A. Baillie, Zhao-Bang Zeng, Peter D. Karp, Oliver Fiehn, Ronald M. Krauss, Rima Kaddurah-Daouk

**Affiliations:** 1 Bioinformatics Research Group, AI Center, SRI International, Menlo Park, California, United States of America; 2 Duke University Medical Center, Durham, North Carolina, United States of America; 3 Genomics Center, University of California, Davis, California, United States of America; 4 Rosa and Company, Cupertino, California, United States of America; 5 Bioinformatics Research Center, NC State University, Raleigh, North Carolina, United States of America; 6 Children's Hospital Oakland Research Institute, Oakland, California, United States of America; Governmental Technical Research Centre of Finland, Finland

## Abstract

Statins are widely prescribed for reducing LDL-cholesterol (C) and risk for cardiovascular disease (CVD), but there is considerable variation in therapeutic response. We used a gas chromatography-time-of-flight mass-spectrometry-based metabolomics platform to evaluate global effects of simvastatin on intermediary metabolism. Analyses were conducted in 148 participants in the Cholesterol and Pharmacogenetics study who were profiled pre and six weeks post treatment with 40 mg/day simvastatin: 100 randomly selected from the full range of the LDL-C response distribution and 24 each from the top and bottom 10% of this distribution (“good” and “poor” responders, respectively). The metabolic signature of drug exposure in the full range of responders included essential amino acids, lauric acid (p<0.0055, q<0.055), and alpha-tocopherol (p<0.0003, q<0.017). Using the HumanCyc database and pathway enrichment analysis, we observed that the metabolites of drug exposure were enriched for the pathway class amino acid degradation (p<0.0032). Metabolites whose change correlated with LDL-C lowering response to simvastatin in the full range responders included cystine, urea cycle intermediates, and the dibasic amino acids ornithine, citrulline and lysine. These dibasic amino acids share plasma membrane transporters with arginine, the rate-limiting substrate for nitric oxide synthase (NOS), a critical mediator of cardiovascular health. Baseline metabolic profiles of the good and poor responders were analyzed by orthogonal partial least square discriminant analysis so as to determine the metabolites that best separated the two response groups and could be predictive of LDL-C response. Among these were xanthine, 2-hydroxyvaleric acid, succinic acid, stearic acid, and fructose. Together, the findings from this study indicate that clusters of metabolites involved in multiple pathways not directly connected with cholesterol metabolism may play a role in modulating the response to simvastatin treatment.

**Trial Registration:**

ClinicalTrials.gov NCT00451828

## Introduction

Statins are HMG-CoA reductase inhibitors that are used to reduce LDL-cholesterol (LDL-C) and, thereby, to reduce CVD risk [1]. However, this class of drugs exhibits a broad spectrum of biological effects that may impact on CVD risk, including improvement of endothelial function by upregulation of endothelial NO synthase (eNOS), decrease in proliferation of vascular smooth muscle cells and macrophages, reduction of platelet activity, stabilization of atherosclerotic plaques, and antioxidant, anti-inflammatory and immunomodulatory effects [2]. In addition several clinically significant side effects have been documented, including myopathy and increased risk for developing Type II diabetes mellitus [3].

Multiple intervention trials with statin drugs have demonstrated a reduction in relative risk for both CVD and stroke. Nevertheless, residual CVD risk remains high and LDL-C response varies greatly. Variation in response to statins can be affected by genetic and environmental influences. Several genetic polymorphisms that contribute to variability in the LDL-C response to statins have been identified [4], but only a small proportion of the variance has been explained by these factors. Additional variables affecting response to statins include diet [5], level of immune response [6], environmental conditions, and drug interactions [7]. Simvastatin is administered as an inactive precursor drug that is activated by endogenous biotransformation pathways. There is increasing interest in the role of gut bacteria in the metabolism of drugs [8], and recent data suggest that secondary bile acids produced by gut microbiome contribute to variation of LDL lowering response to simvastatin [9].

Inter-individual variation in response to statins, and the fact that LDL cholesterol and other biomarkers are not sufficient to predict clinical benefit or side effects, suggest that more reliable biomarkers are needed for identifying the sub-populations that may achieve the most benefit from statin use and those that might be at risk for developing side effects. Metabolomics provides powerful tools for mapping pathways implicated in disease and in response to drug treatment [10], [11]. Sophisticated metabolomic analytical platforms and informatics tools have been developed that have made it possible to define initial signatures for several diseases [12], [13], [14], [15], [16], [17]. Metabolomic “signatures” present in patients who do and do not respond to drug therapy, i.e., signatures that reflect the drug response phenotype, could lead to mechanistic hypotheses that would provide insight into the underlying basis for individual variation in response to drugs such as antidepressants and statins [18], [19], [20].

Previously, using a targeted lipidomics platform, we found that baseline cholesterol ester and phospholipid metabolites were correlated with LDL-C response to treatment in individuals selected from the upper and lower tails of the LDL-C response distribution in the Cholesterol and Pharmacogenetics (CAP) study [21]. C-reactive protein (CRP) response to therapy correlated with baseline plasmalogens, lipids that are involved in inflammation, indicating that distinct metabolic changes are correlated with LDL-C and CRP response to statins. Using a second targeted metabolomics platform in participants from this study, secondary bile acids produced by the gut microbiome were found to be implicated in response to simvastatin [9].

In the present study, we used a non-targeted, broad spectrum pathway agnostic GC-TOF mass spectrometry platform to measure 160 metabolites in 148 CAP study participants and considered the following questions:

1. What is the metabolic signature of exposure to simvastatin?
2. Which elements of the drug signature correlate with LDL-C response?
3. What metabolites at baseline define distinct metabolic profiles (metabotypes) that can distinguish between good and poor response to simvastatin?

## Results

### Analyses in Individuals Sampled From the Full Range of LDL-C Response to Simvastatin

#### Metabolic signature of exposure to simvastatin

**Figure 1:**
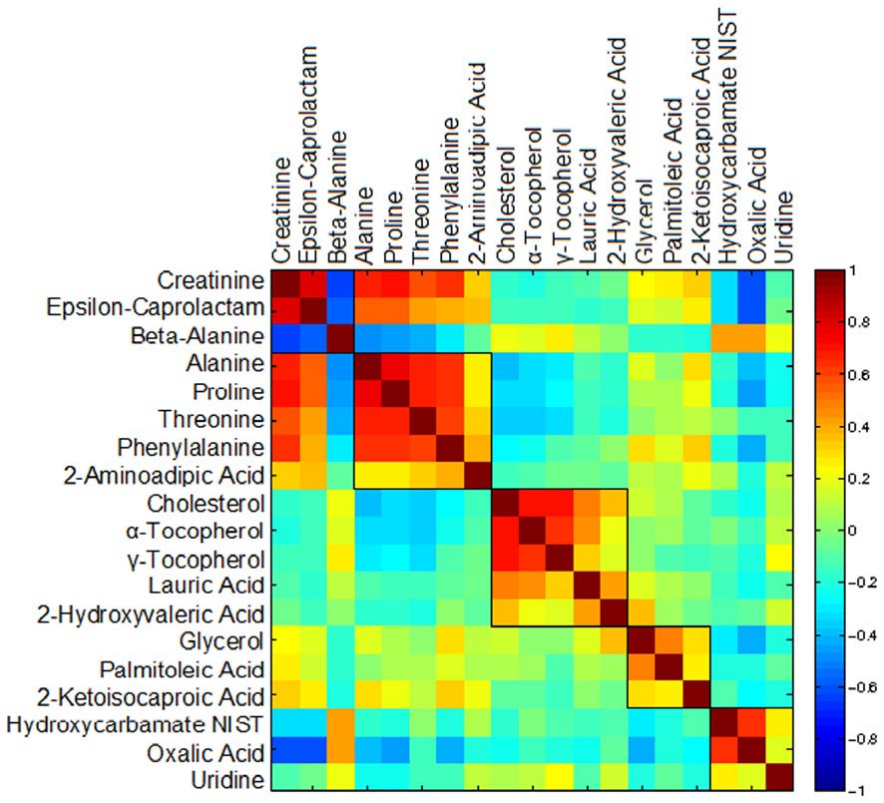
Correlation matrix of metabolites altered by simvastatin in full range participants. Correlations among metabolites in Table 1 were obtained by deriving a Spearman’s correlation coefficient between each pair of metabolites. The color scheme corresponds to correlation strength as shown by the color bar. Red: Better response, more reduction of the metabolite. Blue: Better response, less reduction or increase of the metabolite. The metabolites have been rescaled (divided by the largest absolute value of them) to be clearer on the map. Abbreviations: NIST, National Institute of Standards and Technology.

An untargeted, broad range GC-TOF metabolomics platform was used to profile plasma from 100 participants from across the full range of the distribution of LDL-C response in the CAP study (Table S1). Samples were analyzed before and after six weeks treatment with simvastatin 40 mg/d and Wilcoxon signed rank test was used to define metabolites that significantly changed during treatment (Table 1). Correlations among these metabolites are shown in Figure 1.

**Table 1:** Metabolites significantly altered by simvastatin in full range participants.

Compound	Direction of Change	p-value	q-value
α-tocopherol	decrease	0.0003	0.017
cholesterol	decrease	0.0027	0.055
glycerol	increase	0.0035	0.055
2-hydroxyvaleric acid	decrease	0.0039	0.055
lauric acid	decrease	0.0055	0.055
threonine	increase	0.0060	0.055
oxalic acid	decrease	0.0090	0.069
alanine	increase	0.0100	0.069
phenylalanine	increase	0.0110	0.069
proline	increase	0.0180	0.1
uridine	decrease	0.0200	0.1
2-ketoisocaproic acid	increase	0.0270	0.12
palmitoleic acid	increase	0.0290	0.12
epsilon-caprolactam	increase	0.0300	0.12
hydroxycarbamate^N^	decrease	0.0350	0.13
γ-tocopherol	decrease	0.0370	0.13
2-aminoadipic acid	increase	0.0390	0.13
beta-alanine	decrease	0.0440	0.13
creatinine	increase	0.0450	0.13

N(superscript): indicates a compound identified by spectral matching to the NIST spectral library.

Following simvastatin treatment, a significant decrease was found in plasma levels of cholesterol (p<0.0027, q<0.055), alpha-tocopherol (p<0.0003, q<0.017), and gamma-tocopherol (p<0.037, q<0.13), (vitamin E), and lauric acid (p<0.0055, q<0.055) (Table 1). Changes in levels of these three metabolites were significantly correlated with each other in samples from individuals across the full range of simvastatin response (Figure 1). Changes in cholesterol and lauric acid levels were positively correlated with changes in levels of 2-hydroxyvaleric acid (p<0.0039, q<0.055) (Figure 1). Plasma levels of threonine (p<0.0060, q<0.055), alanine (p<0.010, q<0.069), and phenylalanine (p<0.011, q<0.069), were increased following simvastatin treatment (Table 1). These increases in amino acids were all correlated with reduction in free cholesterol (Figure 1), and were all highly correlated with each other. A pattern of higher levels of essential amino acids appears when considering that 2-ketoisocaproic acid (p<0.027, q<0.12) and 2-aminoadipic acid (p<0.039, q<0.13), intermediates in the degradation of leucine and lysine respectively, were both increased by simvastatin treatment (Table 1). Using the HumanCyc database and the pathway enrichment analysis component of Pathway Tools software, we observed that the metabolites in Table 1 were enriched for the pathway class amino acid degradation with a p-value <0.0032 (data not shown).

#### Changes in metabolites that correlate with LDL-C response to simvastatin

**Figure 2:**
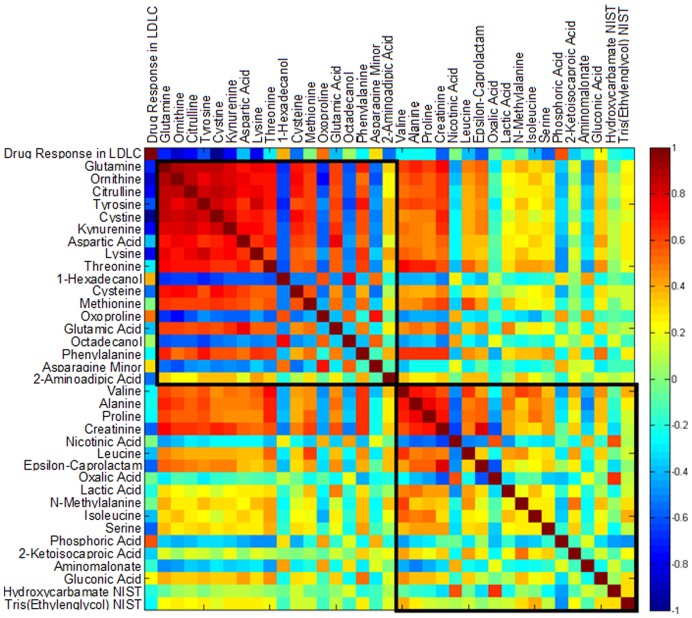
Correlation matrix illustrating two clusters of compounds correlated with simvastatin response in full range participants. The two clusters were identified in a clustering analysis for the change of all metabolites (results not shown) according to their pairwise correlations using the MMC algorithm [14]). Correlations of metabolites to drug response in LDLC were given in the first row and column, and are rescaled (divided by the largest absolute value of them) to be clearer in the map. The color scheme corresponds to correlation strength as shown by the color bar. Red: Better response, more reduction of the metabolite. Blue: Better response, less reduction or increase of the metabolite. Abbreviations: LDLC, Low-Density Lipoprotein Cholesterol; NIST, National Institute of Standards and Technology.

Analysis of the metabolite changes that correlated with LDL-C response in the full range group identified urea cycle intermediates and a group of dibasic amino acids related by shared transporters (Table 2). Specifically, increases in cystine (p<0.0012, q<0.10), ornithine (p<0.0068, q<0.26), lysine (p<0.0100, q<0.26), and citrulline (p<0.0160, q<0.26) were all observed to correlate with the LDL-C lowering effects of simvastatin with significant p-values (<0.05), but high q-values. Individually, these metabolites are not considered statistically significant, but considering the close structural and functional similarities between the first six metabolites correlated to response to simvastatin–we consider this finding compelling from a discovery perspective. Cystine, ornithine, and lysine, the three most significantly correlated metabolites, are all substrates for the cystinuria-related transporter rBAT/B (0, +) AT (SLC3A1/SLC7A9), which mediates uptake of dibasic amino acids at the intestinal and renal membranes. Citrulline and ornithine are also both intermediates in the catabolism of amino acids by the urea cycle and their strong correlation (Figure 2), suggests an increased flux through the urea cycle and amino acid catabolism.

**Table 2:** Metabolite changes significantly correlated with response to simvastatin treatment in full range participants.

Compound	Association with Response	p-value	q-value
cystine	positive	0.0012	0.10
ornithine	positive	0.0068	0.26
lysine	positive	0.0100	0.26
kynurenine	positive	0.0150	0.26
citrulline	positive	0.0160	0.26
glutamine	positive	0.0270	0.37

#### Pre-treatment levels of metabolites correlated with response to simvastatin

We next sought to identify metabolites whose baseline levels were predictive of LDL-C response to simvastatin in the full range group (Table S2). A test of Spearman’s correlation coefficients between post-treatment level of LDL-C and metabolites at baseline adjusting for pre-treatment level of LDL-C showed that greater LDL-C lowering was correlated with lower levels of uridine (p<0.0410, q<0.68) and higher levels of pseudouridine (p<0.0140, q<0.60). While correction for multiple hypothesis testing indicates that these changes are not statistically significant, we report the entire dataset since these metabolites are connected by the enzymatic reaction of pseudouridine synthase.

### Analyses in Good and Poor Responders to Simvastatin

#### Changes in metabolite levels in good and poor responders upon treatment with simvastatin

In good responders we found a decrease in cholesterol (p<0.0022, q<0.18) and an increase in shikimic acid (p<0.0096, q<0.38) (Table S3). Shikimate is an indole precursor of phenylalanine, tyrosine and tryptophan, produced by plants and bacteria, but not animals, suggesting increased synthesis by the gut microbiome and/or increased transport of shikimic acid across intestinal membranes. While below the level of statistical significance, we observed increases in fructose and the sugar acids pentonic acid and hexaric acid that correlated with change in LDL-C in the good responder group (data not shown) and were opposite to the effects seen for these metabolites in poor responders (Table 3).

**Table 3:** Metabolites significantly altered by simvastatin in poor responders.

Compound	Direction of Change	p-value	q-value
glycolic acid	decrease	0.0005	0.039
fructose	decrease	0.0012	0.039
glucose	decrease	0.0020	0.039
pentonic acid*	decrease	0.0020	0.039
ethanolamine	decrease	0.0072	0.11
1-hexadecanol	decrease	0.0130	0.16
hydroxylamine	decrease	0.0180	0.2
threonic acid	decrease	0.0250	0.24
pseudo uridine	decrease	0.0310	0.24
pelargonic acid	decrease	0.0340	0.24
pentonic acid*	decrease	0.0370	0.24
succinic acid	decrease	0.0390	0.24
hexaric acid*	decrease	0.0420	0.24
α-mannosylglycerate	decrease	0.0420	0.24
uric acid	decrease	0.0460	0.24

* indicates a partially identified compound: pentonic acid is an aldonic acid with five carbons and hexaric acid is an aldonic acid with six carbons.

While metabolomics measurements detected no reduction in plasma cholesterol levels in poor responders (an independent verification of clinical measurements) several metabolites did change significantly in the poor responder group upon treatment with simvastatin (Table 3). In poor responders, simvastatin induced a reduction in plasma levels of the sugars fructose (p<0.0012, q<0.039) and glucose (p<0.0020, q<0.039)–the latter strongly correlated with reductions in alpha-mannosylglycerate (0.809, p<0.0001) and pseudouridine (0.704, p<0.0001) (data not shown). Glycolic acid decreased significantly (p<0.0005, q<0.039), and this decrease was correlated in the full range group with a decrease in hydroxylamine (0.598, p<.002)(not shown). Together, these clusters of metabolites may provide a more robust biomarker for simvastatin non-response than a single changed metabolite.

#### Baseline metabolites that are significantly different between good and poor responders

We detected several metabolites with highly significant correlations between pre-treatment levels and LDL-C response to simvastatin in the good responder group (Table 4). Lower baseline levels of xanthine (p<0.0001, q<0.00068), 2-hydroxyvaleric acid (p<0.0001, q<0.0013), succinic acid (p<0.0009, q<0.017) and stearic acid (p<0.0039, q<0.037) were all significantly correlated with greater LDL-C response. Conversely, higher pretreatment level of galactaric acid (hexaric acid) (p<0.0034, q<0.037) was correlated with increased responsiveness to simvastatin.

**Table 4:** Metabolites with baseline levels significantly different between good and poor responders among extreme range participants.

Compound	Association with Response	p-value	q-value
xanthine	negative	<0.0001	0.00068
2-hydroxyvaleric acid	negative	<0.0001	0.0013
succinic acid	negative	0.0009	0.017
stearic acid	negative	0.0032	0.037
hexaric acid*	positive	0.0034	0.037
heptadecanoic acid	negative	0.0260	0.19
pelargonic acid	negative	0.0300	0.19
4-hydroxyproline	negative	0.0300	0.19
capric acid	negative	0.0320	0.19
oxoproline	negative	0.0360	0.19
pentonic acid*	negative	0.0410	0.21
fructose	negative	0.0460	0.21

* indicates a partially identified compound: pentonic acid is an aldonic acid with five carbons and hexaric acid is an aldonic acid with six carbons.

#### Multivariate analysis of metabolomic profiles of good and poor responders at baseline

**Figure 3:**
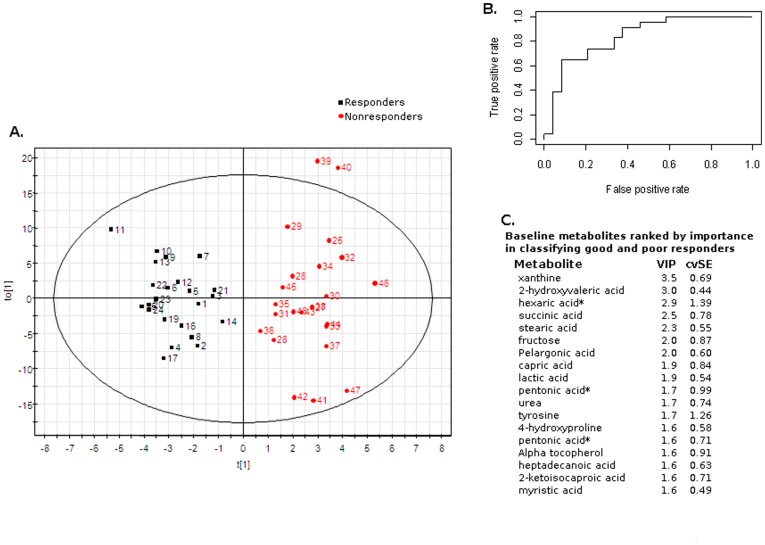
OPLSDA of baseline metabolites classifies good and poor responders. (A) Orthogonal partial least square discriminant analysis was used to classify good and poor responders based on log-transformed baseline concentration of metabolites (R^2^ = 0.87, Q^2^ = 0.31). Good responders are shown in black and poor responders in red. Baseline metabolites were log-transformed and normalized (described in methods). Performance evaluation by 7-fold cross validation yielded the following statistics: prediction accuracy: 74%; sensitivity: 70%; specificity: 79% (not shown). (B) ROC curve of true positive rate (x-axis) versus false positive rate (y-axis) yields an area under the curve (AUC) of 0.84. (C) Baseline metabolites ranked by importance in classifying good and poor responders in the OPLS model. *indicates a partially identified compound: pentonic acid is an aldonic acid with five carbons and hexaric acid is an aldonic acid with six carbons. Abbreviations: VIP, variable importance score; cvSE, standard error derived from cross validation.

Orthogonal partial least square discriminant analysis (OPLSDA) was used to compare baseline levels of metabolites in good and poor responders (Figure 3). The results shown in Figure 3A are for a three-component model with 2 orthogonals that was built on all the good and poor responders. The model yielded a R^2^ of 0.87 and a Q^2^ of 0.31. Furthermore, 7-fold cross validation was then used to better evaluate the prediction of drug response. Specifically, in each round of cross validation, about 6/7 of the subjects were used to build a three component OPLSDA model, which was then used to predict the drug response for the remaining 1/7 of the subjects. It was found that on average the OPLSDA models achieved a prediction accuracy of 74% with 70% sensitivity and 79% specificity. The ROC curve of true positive rate vs false positive rate yields an area under the curve (AUC) of 0.84 (Figure 3B). In general, diagnostic testing indicates that modeling of these metabolites yields a robust predictive tool for distinguishing between good and poor responders. Metabolites that contribute to their separation are shown in Figure 3C, with variable importance scores (VIP) and cross validation standard errors (cvSE). Top metabolites that correlate with separation of the groups include xanthine (VIP: 3.5, cvSE: 0.69), 2-hydroxyvaleric acid (VIP: 3.0, cvSE: 0.44), succinic acid (VIP: 2.5, cvSE: 0.78), stearic acid (VIP: 2.3, cvSE: 0.55) and fructose (VIP: 2.0, cvSE: 0.87). Additional metabolites of unknown identity also contributed to the model separating good and poor responders classes (data not shown). This modeling supports the hypothesis that individuals in the tails of the response distribution in the CAP study comprise metabolically distinct subgroups and that their metabolic profiles (metabotypes) might contribute to their differing responses.

## Discussion

In this study a mass spectrometry-based metabolomics platform (GC-TOF) that detects small molecules below 550 Da in a broad spectrum of chemical classes and metabolic pathways was used to map global metabolic effects of simvastatin. An untargeted metabolomics approach can yield many unrelated findings; but the dataset reported here has some striking patterns. In broadly sampled full range responders, we have detected an increase in amino acids, specifically essential amino acids and their degradation products. An increase in dibasic amino acids was correlated with response to simvastatin, again not a random set of amino acids, but specifically those transported by the cystinuria and arginine transporters. In poor responders we detected a decrease in plasma fructose, glucose and sugar acids that was not seen in good responders. This combination of findings may suggest a distinct pattern of energy usage between poor responders and the broader spectrum of response phenotypes. Poor responders could also be separated from good responders using an OPLS-DA model of baseline metabolites. Together these findings suggest that metabolomics analysis might be developed for defining a population unlikely to benefit from statin use sufficient to warrant risk of the spectrum of side-effects.

A more detailed analysis of the GC-TOF data reveals changes in a diverse set of compounds, some anticipated and some unexpected. The expected changes included decreased free cholesterol in individuals selected from both the full range of LDL-C response (Table 1) and those selected from the 10% tail of good responders (Table S2), but not from the poor response tail (Table 3). The very strong correlation of cholesterol with alpha and gamma tocopherol (vitamin E) may reflect the close relationship between tocopherols and cholesterol metabolism. In addition to their antioxidant effects, alpha tocopherol modulates LDL receptor concentrations, and reduces CD36 receptor and ABC1 transporter expression [22], [23], [24], [25]. Simvastatin has been shown previously to reduce alpha-tocopherol levels in plasma following both short-term [26] and long-term exposure [27]. In studies where simvastatin treatment was augmented with alpha-tocopherol, increased lowering of total and LDL cholesterol was observed [28], indicating that lower alpha-tocopherol levels are not mediating the LDL-C reductions. It has been reported that simvastatin treatment increases the capacity of LDL particles to transport lipid soluble antioxidants [27] and oxygenated and hydrocarbon carotenoids [29], suggesting that the statin-induced reductions in total plasma levels of tocopherol which we observed are accompanied by increased tocopherol content of LDL particles, and hence the net result may contribute to greater resistance of these particles to oxidative stress, and hence to reduced atherogenic potential. Additional pleiotropic effects of simvastatin might include an up regulation of triacylglycerol lipase activity (EC# 3.1.1.3, LIPC) [30]. This might explain the increase in plasma levels of glycerol that we detect in our analysis (Table 1).

The increase in creatinine and modulation of several amino acids with simvastatin treatment (Table 1) was associated with strong correlations among those metabolites (Figure 1) suggesting that a common metabolic pathway was affected. We have also observed that levels of shikimic acid, an enterobacteria-derived precursor of aromatic- and indole-containing amino acids, are also altered by statin treatment in the good responders (Table S2).

Pseudouridine, the C-glycoside isomer of the RNA nucleotide uridine, is present in essentially all ribosomal RNAs and seems to be affected by simvastatin. In humans pseudouridine synthase converts uridine to pseudouridine, which is a dead-end metabolite and generally excreted in urine. An increased level of pseudouridine suggests a decrease in the rate of excretion, but together with a reduced level of uridine, a more likely scenario is an increase in the activity of pseudouridine synthase.

The inter-correlated changes in several amino acids and their degradation products and the correlation between changes in these amino acids and LDL-C change (Figure 2) point to possibly important functions of amino acids in simvastatin mechanism of action. Our data indicate a statin-induced change in amino acid degradation or transport. Our pathway analysis indicates that metabolites that changed in the full range participants in response to simvastatin (Table 1) were enriched for the pathway class amino acid degradation. In the full range participants, metabolites that correlate with LDL-C response to simvastatin (Table 2) included dibasic amino acids that are substrates for the cystinuria-related transporter, potentially indicating changes in transport. In addition, the changes in citrulline and ornithine suggest an increased flux through the urea cycle (Figure 2, Table 2) again pointing to a change in amino acid degradation. Since simvastatin treatment either has no effect or can improve kidney function [31], [32], [33] it is unlikely that the changes in amino acids were due to renal dysfunction.

The previously published identification of amino acid metabolism as a marker of changes in cholesterol metabolism is consistent with the development of peptide biomarkers of cardiovascular disease [34]. In addition, amino acids have been identified as predictors of the risk of developing diabetes and possibly contributing to changes in insulin sensitivity [16], [35].

The metabolite most significantly different between good and poor responders at baseline was the purine metabolite xanthine, which is implicated in oxidative stress cascades (Table 4). Xanthine is the final breakdown product of purine nucleotides and the immediate precursor of uric acid. Xanthine is the substrate of xanthine oxidase, which produces hydrogen peroxide and hence is implicated in mechanisms of oxidative stress. Since free radicals are known to decouple NOS enzymatic activity, a lower basal level of xanthine and purine degradation may yield an environment for more robust NOS signaling. Xanthine oxidase inhibitors are an emerging treatment for cardiac ischemia [36], [37] and xanthine oxidase has been considered as a therapeutic target for cardiovascular disease [38]. Together, these results suggest that we have identified a metabolite related to oxidative stress that is robustly correlated to LDL response to simvastatin. Furthermore, recent results indicate that hyperuricemia resulting from increased purine degradation attenuates NO production by inhibiting arginine transport at the NOS associated CAT-1 transporter [39]. It has not escaped our attention that statin effects on arginine transport function may contribute to therapeutic effects on endothelial function by increasing NOS activity.

Recent studies employing *ex vivo* cultures of intestinal bacteria to metabolize simvastatin have revealed a degradation of drug, producing a series of metabolites including and similar to 2-hydroxyvaleric acid (2-hydroxypentanoic acid) [40]. We detected 2-hydroxyvaleric acid as a significantly decreased metabolite following *in vivo* treatment with simvastatin (Table 1). Since this metabolite is present in simvastatin naïve samples and is decreased by simvastatin treatment, it is possible that simvastatin, or a metabolite of simvastatin, inhibits an enzyme that produces 2-hydroxyvaleric acid. Conversely, low basal levels of 2-hydroxyvaleric acid exhibit a highly significant correlation to LDL-C reduction in good and poor responders (Table 4). This suggests lower activity of a promiscuous enzyme that produces 2-hydroxyvaleric acid, and perhaps metabolizes simvastatin, might also result in lower rates of simvastatin degradation and differential pharmacokinetics. This observation, as well as those described for shikimic acid above, suggests that variation in gut microbiome activity can contribute to mechanism of variation in response to simvastatin (Table S2). An influence of gut micoflora on simvastatin response was also suggested by our recent finding that several secondary bile acids derived from microbial metabolism are predictive of LDL-C response to simvastatin in the CAP study [9]. This suggests that genetic, gut microbiome and environmental interactions might all contribute to mechanism of variation of response to simvastatin.

While we consider this study a discovery project to validate the experimental paradigm and elucidate pleiotropic features, the sample size is commensurate with other metabolomics studies. However, larger studies will be necessary to validate clinical utility–which will ideally define pre-treatment metabolic signatures that are predictive of efficacy of simvastatin as well as other statins in reducing clinical cardiovascular events, as well as those that can identify individuals most likely to experience adverse effects of treatment. Further, by assessing such markers, the design of future clinical trials can be improved by excluding individuals who are least likely to derive clinical benefit. The observation that people selected from the ends (best and worst responders) are biochemically distinct suggests that metabolomics might be a powerful tool for sub classification of individuals as an important step in streamlining clinical trials and personalizing treatment. Finally, the application of both mass spectrometry and lipidomics platforms in the CAP study has demonstrated the value of this approach in mapping global metabolic effects of simvastatin and in highlighting the pathways that may modulate variation in its clinical efficacy and in its multiple biological effects. We have undertaken this systems biology approach to define pharmacometabolomic changes to generate hypotheses of pleiotropic mechanisms mediating simvastatin effects. These results suggest that functional studies are warranted into the interaction of simvastatin with endogenous and enterobiome metabolites at bile and amino acid transporters.

## Materials and Methods

### Clinical Samples

Plasma samples were analyzed from participants in the Cholesterol and Pharmacogenetics (CAP) study, a trial in which 944 Caucasian and African-American men and women with total cholesterol levels of 160–400 mg/dL were treated with simvastatin at 40 mg per day for six weeks (Table S1). Information about the participants selected for this study has been published previously. Briefly, this study was designed to examine genetic and non-genetic factors affecting the response to simvastatin therapy in healthy, drug-naïve patients [21]. Participants were followed for a total of 6 weeks on simvastatin therapy (40 mg at bedtime) and were seen at clinic visits conducted at two-week intervals. Blood specimens from each participant were obtained after an overnight fast at the screening visit, after a 2-week placebo run-in (enrollment visit), and following 4 and 6 weeks of simvastatin administration. Samples used in this study were collected at baseline and at 6 weeks of therapy. Medication compliance was assessed by pill count every two weeks and averaged over 95%. Overall, treatment with simvastatin lowered low-density lipoprotein (LDL) cholesterol by 54 mg/dl and increased high-density lipoprotein (HDL) cholesterol by 2 mg/dl. The magnitude of the lipid and lipoprotein responses, however, differed among participants according to a number of phenotypic and demographic characteristics [23]. Data on dietary intake was not collected, but subjects were instructed not to change their diet. No minors took part in this study. Approval for this study of the analyses of determinants of simvastatin response in the CAP study was granted by the Children's Hospital and Research Center Institutional Review Board, University of California San Francisco Committee on Human Research, and University of California Los Angeles Office of the Human Research Protection Program and written informed consent was obtained from all participants. The research was conducted in accordance with the Declaration of Helsinki.

Two subgroups of participants were selected: the first from the extreme range of response, ‘good and poor responders’, consisted of 24 individuals selected from the top 10% of the LDL-C response distribution who were matched for body mass index, race, and gender to 24 individuals in the lowest 10% of responders, with response to therapy defined as the percentage change in LDL cholesterol from baseline. The second set ‘full range’ was 100 individuals randomly selected from the entire CAP study, excluding participants who had been selected for the extreme range group. These full range participants are representative of the population for age, race, gender, and BMI. On the other hand, the extreme range subset provides a means to explore the differences in metabolite profiles and predictors of the highest and lowest responses to drug therapy. Metabolomic analyses of statin-induced changes in bile acids in both groups [9] and the major lipid classes in the full range participants have been reported recently [19].

### GC-TOF Mass Spectrometry Analysis

Plasma samples (30 µl) were extracted and derivatized as reported previously [41]. Briefly, 15 µl aliquots were extracted by 1 ml of degassed acetonitrile:isopropanol:water (3∶3:2) at –20°C, centrifuged and decanted with subsequent evaporation of the solvent to complete dryness. A clean-up step with acetonitrile/water (1∶1) removed membrane lipids and triglycerides and the supernatant was dried down again. Internal standards C8–C30 fatty acid methyl esters (FAMEs) were added and the sample was derivatized by methoxyamine hydrochloride in pyridine and subsequently by N-methyl-trimethylsilyltrifluoroacetamide (MSTFA) (1 ml bottles, Sigma-Aldrich) for trimethylsilylation of acidic protons.

A Gerstel MPS2 automatic liner exchange system was used to inject 1 µl of sample at 50°C (ramped to 250°C) in splitless mode with 25 s splitless time. An Agilent 6890 gas chromatograph (Santa Clara CA) was used with a 30 m long, 0.25 mm i.d. Rtx5Sil-MS column with 0.25 µm 5% diphenyl film and additional 10 m integrated guard column was used (Restek, Bellefonte PA). Chromatography was performed at constant flow of 1 ml/min ramping the oven temperature from 50°C for to 330°C with 22 min total run time. Mass spectrometry was done by a Leco Pegasus IV time of flight mass spectrometer with 280°C transfer line temperature, electron ionization at −70 V and an ion source temperature of 250°C. Mass spectra were acquired from m/z 85–500 at 20 spectra s^-1^ and 1750 V detector voltage. Result files were exported to our servers and further processed by our metabolomics BinBase database [42]. All database entries in BinBase [41] were matched against the Fiehn mass spectral library of 1,200 authentic metabolite spectra using retention index and mass spectrum information or the NIST05 commercial library. Identified metabolites were reported if present with at least 50% of the samples per study design group (as defined in the SetupX database) [43]. Quantitative data were normalized to the sum intensities of all known metabolites and used for statistical investigation.

### Statistical Analyses

The Wilcoxon signed rank test was used to evaluate lipid changes in metabolites pre and post treatment. For relationships between changes in metabolite and statin response of LDL-C, metabolite changes were defined as post-treatment level minus pre-treatment level. For the 100 randomly selected full range participants, metabolite changes were correlated with statin response of LDL-C by calculating their Spearman’s correlation coefficients with post-treatment level LDL-C, adjusting for pre-treatment level of LDL-C. The adjusted Spearman’s correlation is equivalent to the Pearson’s correlation between the residuals of the linear regression of the ranks of post-treatment level of LDL-C and the metabolite change on the ranks of pre-treatment level of LDL-C. To associate metabolite changes with statin response of LDL-C using good and poor responder data, the difference of metabolite changes between good responders and poor responders using the Wilcoxon rank sum test was tested. Those significant metabolites are the compounds whose change is associated with statin response of LDL-C.

For predicting response of LDL-C to simvastatin, baseline metabolite levels were correlated with statin response of LDL-C by calculating their Spearman’s correlation coefficients with post-treatment level of LDL-C, adjusting for pre-treatment level of LDL-C using the same aforementioned method. In order to associate metabolites at baseline to statin response in LDL-C using extreme subjects’ data, the difference in metabolites at baseline between good responders and poor responders was tested using the Wilcoxon rank sum test. For all the above univariate tests, q-values [44] were calculated for controlling multiple testing false discovery rate.

Correlations among metabolites were obtained by deriving Spearman’s correlation coefficient between each pair of metabolites. Correlation matrixes were used to visualize the correlation between metabolites and drug responses. The modulated modularity clustering algorithm is used to cluster metabolites based on their pairwise Spearman’s correlation coefficients [45].

Orthogonal partial least square discriminate analysis (OPLSDA) was used to classify good and poor responders. OPLSDA is built on OPLS [46], a modification of the widely used PLS method. Compared to PLS, OPLS is able to separate variation in metabolomics data into two parts that are related or unrelated to the drug response phenotype, which facilitates the interpretation of results with preserved prediction ability [46]. The OPLSDA model was built on log-transformed and then normalized baseline concentration of metabolites. Normalization was performed by subtracting each metabolite by its sample mean and dividing that by its standard error. The software SIMCA-P+ version 12.0 was used to perform the analysis. Two orthogonal components were included in the model. A variable importance (VIP) score is used to rank metabolites’ contribution in separating the two response groups. Prediction diagnostics were derived from 7-fold cross validation. Each fold contains balanced numbers of good and poor responders.

## Supporting Information

Table S1 **Demographics of CAP patients included in the current GC-TOF metabolomics study.** The table lists the comparison of the full-range, good and poor responder subgroups used in this study based on age, gender, race, BMI, change in LDL-C and basal levels of LDL-, HDL- and total cholesterol.
(DOC)

Table S2 **Metabolites for which baseline levels were significantly correlated with response to simvastatin in full range participants.** The table shows the association of pre-treatment levels of gluconic acid, pseudouridine, maltose, leucine and uridine to the amount of change in LDL-C after simvastatin administration. Metabolites listed are significantly correlated to response to simvastatin based on p-values, but not following correction for false-discovery rate (q-values).
(DOC)

Table S3 **Metabolites significantly altered by simvastatin in good responders among extreme range participants.** The table shows the direction of change in cholesterol, shikimic acid and ethanolamine following simvastatin administration. Metabolites listed are significantly altered by simvastatin based on p-values, but not following correction for false-discovery rate (q-values).
(DOC)
