# Supplementary material for: Metabolomics Reveals Amino Acids Contribute to Variation in Response to Simvastatin Treatment
Source: PLoS One. 2012 Jul 9;7(7):e38386. doi: 10.1371/journal.pone.0038386 (PMC3392268; doi:10.1371/journal.pone.0038386)
Supplement: Table S1 — Demographics of CAP patients included in the current GC-TOF metabolomics study. The table lists the comparison of the full-range, good and poor responder subgroups used in this study based on age, gender, race, BMI, change in LDL-C and basal levels of LDL-, HDL- and total cholesterol. (DOC) [file pone.0038386.s001.doc]

**Table S1.** **Demographics of CAP patients included in the current GC-TOF metabolomics study.**

| **Selected Participants** | **Extreme Range** | | **Full Range** |
| --- | --- | --- | --- |
|  | **Good Responders**  **n=24** | **Poor Responders**  **n=24** | **All Participants**  **n=100** |
|  | **%** | **%** | **%** |
| Male (%) | 33 | 33 | 48 |
| Race (% African American) | 25 | 29 | 30 |
|  | **mean ± SD** | **mean ± SD** | **mean ± SD** |
| Body Mass Index (kg/m^2^) | 28.5 ± 6.4 | 29.1 ± 5.1 | 28.5 ± 5.1 |
| Decrease in LDL-C (%) | 63.06 ± 4.2 | 7.62 ± 9.4 | 41.4 ± 11.7 |
| Age (years) | 60.3 ± 13.8 | 53.1 ± 12.4 | 53.6 ± 12.6 |
| Initial LDL-C (mg/dL) | 133.2 ± 31.9 | 120.1 ± 29.6 | 138.4 ± 35.5 |
| Initial HDL-C (mg/dL) | 58.17 ± 15.46 | 53.66 ± 18.57 | 52.6 ± 16.5 |
| Initial Total Cholesterol (mg/dL) | 219.58 ± 39.55 | 201.75 ± 32.71 | 215.8 ± 39.2 |
