# Supplementary material for: Metabolomics Reveals Amino Acids Contribute to Variation in Response to Simvastatin Treatment
Source: PLoS One. 2012 Jul 9;7(7):e38386. doi: 10.1371/journal.pone.0038386 (PMC3392268; doi:10.1371/journal.pone.0038386)
Supplement: Table S2 — Metabolites for which baseline levels were significantly correlated with response to simvastatin in full range participants. The table shows the association of pre-treatment levels of gluconic acid, pseudouridine, maltose, leucine and uridine to the amount of change in LDL-C after simvastatin administration. Metabolites listed are significantly correlated to response to simvastatin based on p-values, but not following correction for false-discovery rate (q-values). (DOC) [file pone.0038386.s002.doc]

**Table S2**. **Metabolites for which baseline levels were significantly correlated with response to simvastatin in full range participants.**

| **Compound** | **Association with Response** | **p-value** | **q-value** |  |
| --- | --- | --- | --- | --- |
| gluconic acid | positive | 0.0066 | 0.55 |  |
| pseudouridine | positive | 0.0140 | 0.60 |  |
| maltose | positive | 0.0240 | 0.66 |  |
| leucine | negative | 0.0320 | 0.67 |  |
| uridine | negative | 0.0410 | 0.68 |  |
