# Supplementary material for: Metabolomics Reveals Amino Acids Contribute to Variation in Response to Simvastatin Treatment
Source: PLoS One. 2012 Jul 9;7(7):e38386. doi: 10.1371/journal.pone.0038386 (PMC3392268; doi:10.1371/journal.pone.0038386)
Supplement: Table S3 — Metabolites significantly altered by simvastatin in good responders among extreme range participants. The table shows the direction of change in cholesterol, shikimic acid and ethanolamine following simvastatin administration. Metabolites listed are significantly altered by simvastatin based on p-values, but not following correction for false-discovery rate (q-values). (DOC) [file pone.0038386.s003.doc]

**Table S3. Metabolites significantly altered by simvastatin in good responders among extreme range participants.**

| **Metabolite** | **Direction of Change** | **p-value** | **q-value** |
| --- | --- | --- | --- |
| Cholesterol | decrease | 0.0022 | 0.18 |
| skikimic acid | increase | 0.0096 | 0.38 |
| ethanolamine | decrease | 0.0180 | 0.47 |
